# British Gynæcological Society

**Published:** 1889-06

**Authors:** 


### BRITISH GYNÆCOLOGICAL SOCIETY.

*Meeting Wednesday, April 10, 1889.*

### GENERAL FIBROID DEGENERATION.

Dr. R. T. Smith showed a specimen of unusual interest, consisting of the contracted stomach, ovaries, and uterus removed from a patient, and showing changes of great rarity. There was fibroid thickening of the stomach, intestines, peritoneum, ovaries, and pelvic viscera, and the case had a peculiar interest from a clinical point of view, because the patient came to the hospital with a large abdominal swelling, which naturally caused the question of operation to be discussed. It also raised the question of the remote consequences of chronic pelvic inflammation. The patient was thirty-five years of age. She had been married twelve years, and had one child ten years previously. For four years she had been getting larger in the body, and complained of smarting pain in the abdomen. About a year ago the appetite began to fail, and vomiting occurred twice or three times a week. Then the symptoms became such that she had to keep her bed. For the last four months the navel had protruded to the size of a nut, and was tender. Menstruation was normal. She had night-sweats and cough, and was very much emaciated. She said she had always been a "bilious" subject, and after her confinement had an attack of inflammation, which kept her in bed for a month. On admission the abdomen was uniformly distended, and measured 33 inches at the umbilicus. The most noticeable feature after the abdominal distention was the varying resonance, which moved slowly as she changed position. The diagnosis lay between malignant disease of the omentum and rupture of a cyst. On deep palpation masses could be felt. The uterus was fixed in all directions, and the anterior cul-de-sac was occupied by a dense elastic swelling. On November 27, in consequence of the severity of the vomiting, she was tapped, and seven and a half pints of fluid were drawn off, the first part of which was clear, but the latter part like soup. Then the diagnosis of omental cancer seemed fixed. She was tapped again on January 10, and died a fortnight later, after very severe vomiting, which presented this curious feature, that she rejected the first part of a meal, but retained the rest. *Post mortem* it was found that the liver and spleen were normal, except as regards the peritoneum, which was greatly thickened throughout, was white and opaque, and was generally granular. All the other organs were contracted by the growth of fibrous tissue, and the small intestines were crowded together into a mass. The peritoneal cavity contained turbid serum. The stomach was greatly reduced in size, chiefly by fibroid degeneration of the sub-mucosa. The uterus was fixed in acute ante-flexion. The ovaries were larger than normal, and were firmly fibrous. Microscopical examination showed that there was universal thickening of the sub-peritoneal connective tissue, due to fibroid changes. He observed that the condition was one as to which very little was known, although Fagge refers to a case which was the exact parallel of his own, and that author suggested that the condition might be associated with chronic pelvic inflammation.

Dr. Bedford Fenwick mentioned that he had ransacked the records of the Pathological Society, but he had not been able to come across anything at all like it. He would move that the specimens be referred to the pathological sub-committee.

The motion was seconded by Mr. Tait, and was agreed to unanimously.

Dr. Routh asked whether the condition had anything to do with what he was disposed to call an epidemic of fibroid? He had never heard so much about fibroid in women as at present, and as cancer had increased in frequency, he asked whether fibroids had done so also, and if so, wherefore?

Dr. Travers asked whether there was any history of syphilis or spirit drinking?

Dr. Rutherford asked whether there was any tubercular history?

Dr. Smith, in reply, said that for four years, the patient had been subject to vomiting and pain after eating. There was no history of syphilis nor spirit drinking.

FIBROID TUMORS OF OVIDUCT.

Dr. Heywood Smith showed two specimens of considerable interest. One was of fibroid tumors of the oviduct removed from a lady, æt. 32, married ten years, no children. She had been seen by Professor Wallace, of Glasgow, four years ago, and he had found some swelling in the neighborhood of the ovaries. He operated on the 22nd March last. The left ovary was evidently dermoid. The oviduct was enlarged towards the fimbriated extremity, and was distended with inspissated pus. The ovary on the right side was perfectly healthy, but the oviduct exhibited that rare condition of fibroid tumor, of which there were several. The patient did well, and was up and about in little over a fortnight.

Mr. Lawson Tait thought at first that what Dr. Smith had taken to be fibroid growth was in reality cheesy pus contained in a thickened tube, but on further examination withdrew this remark.

Dr. Routh suggested that this specimen also should be referred to the pathological sub-committee for examination and report, and this was agreed to.

Dr. Bantock said it appeared to him to be an exaggerated condition of what they called chronic salpingitis, without any accumulation in the tube.

ABSENCE (?) OF OVIDUCTS.

Dr. Heywood Smith then showed the appendages removed from a young married woman. She had been married for nearly seven years, never had a child, but was said to have had four miscarriages, the last two years ago. She began to menstruate at ten and a half. Since her marriage she had had fits of hystero-epilepsy, as many as nine or ten in a day. Then she had brain fever for seven months, with delirium, and later on was laid up for three months after her supposed second abortion. There was no flexure of the uterus, which, however, was shunted to the right of the middle line. It measured two and a half inches, and to the left there was a cystic swelling. The remarkable thing in the specimen was that he could find no trace of the oviducts, and he asked the opinion of the Fellows as to whether they were dilated and folded down on the ovaries.

Mr. Lawson Tait said that either there were no tubes to remove, or they must have been left behind by Dr. Smith. He was inclined to take the former view. He observed that it was perfectly possible to get completely developed ovaries and no tubes at all.

Dr. Barnes said he thought that the statements as to miscarriages ought not to be accepted unless the remains of the ovum had been seen. Decidua might be passed with blood after a suspension of menstruation, and give rise to the impression that a miscarriage had taken place. On the suggestion of Dr. Heywood Smith this specimen was also referred to the subcommittee for examination and report.

CASE OF EROTIC FEELINGS PRECEDING AND FOLLOWING EPILEPSY.

Dr. Routh read the notes of a case in which erotic symptoms preceded epilepsy. The patient was a lady whom he had first seen in 1874. She was then unmarried, and was suffering at that time from leucorrhea, with ulceration of the cervix and follicular vaginitis, also dysmenorrhœa. She complained at times of something very much like fits. She was markedly hysterical. She had been addicted to habits of masturbation. She was now married, and had had three children. Since her marriage the fits had recurred, and sometimes followed conjugal relations. They were always stayed by valerian and the bromides. He read his notes of the case on October 18th. The catamenia had been unusually copious, but had lately been very irregular, and finally stopped, and headache and symptoms of confusion of intellect had followed. She was then forty-five. The uterus was very large, reaching above the umbilicus. The passage of a sound into the uterus was very painful and brought on a fit. He ultimately decided to try the effects of the negative electric current to reinduce a menstrual flow, but on the occasion on which he did this she developed strong erotic symptoms, and had an epileptiform attack, after which she again became aggressively amorous to a lady to whose care she was for two hours consigned. The author pointed out that these were precisely the circumstances under which charges were made against medical men, and though his long standing relations with this particular patient prevented anything of the kind, in that case, it was evident that the situation was full of danger under other circumstances.

Dr. Hills said it would be a great advantage to fellow practitioners if the details of this case be consigned in the "Transactions," so that it might be available on any future occasion.

Mr. Tait referred to the well known case of Dr. Bradley. He said that it was important to notice the association of a certain form of epilepsy with an erotic condition. These things ought to be widely known. They ought to be in our text-books, and men should not be sent to prison on charges made by women who were very likely to be suffering from sexual disturbances of this kind. He added that another curious point was charges brought by children under the new law, He had been engaged by the police to investigate these cases, and in only one out of twenty or thirty was there reason to believe that such an offense had been committed.

Dr. Barnes said it was important that this case should be put on record, and mentioned a case in his own experience, insisting upon the necessity of inquiring into these statements very carefully.

Dr. Heywood Smith said that the relation of the electric current to the production of these feelings ought to be cleared up.

Mr. Tait said that one would like to know from authorities on epilepsy how far the erotic excitement was the cause of the epilepsy?

Dr. Heywood Smith pointed out that in the case he had just brought forward, the patient had no fits before marriage, but afterwards had seven or eight in a day.

Dr. Bedford Fenwick insisted upon the importance of remembering the connection that existed between erethism and mental trouble.

Dr. Purcell asked whether there was any epileptic history in Dr. Routh's patient?

The President said the application of the current to the interior of the uterus was not likely to give rise to erotic feelings. The cervix was sensitive, and that might be protected. He asked whether the woman in Dr. Routh's case had expressed the opinion that liberties had been taken with her. He thought that gynæcologists were in a better position than most practitioners since patients came to them expressly to have their reproductive organs examined.

Dr. R. T. Smith said that he had been consulted only that week in reference to a young lady, aged 23, who had had fits, consequent on masturbation.

Dr. Routh, in reply, said his only object in bringing the case before the Society was to place on record a well authenticated case in which erotic symptoms preceded, and followed epileptoid attacks. It was for the same reason that he read his paper on Nymphomania in order to show the absurdity of certain charges made. He observed that even an ordinary vaginal examination was sufficient in some women to provoke the sexual organs, therefore it would not apply to electricity only. The patient had not accused him of having taken liberties, but she had retained her impressions, and had expressly authorized him to publish the case because she felt that other people under similar circumstances might bring a charge against medical men.

